# Parents’ precarious work schedules and children’s asthma management

**DOI:** 10.1186/s12889-026-26274-y

**Published:** 2026-02-09

**Authors:** Kelly Quinn, Katerine Perez, Daniel Schneider

**Affiliations:** 1 Department of Sociology, University of California, Berkeley, Berkeley, CA USA; 2 Department of Sociology, Columbia University, New York, New York USA; 3 Harvard Kennedy School, Harvard University, Cambridge, Massachusetts USA

**Keywords:** Asthma, Precarious work, Scheduling

## Abstract

**Background:**

Many working parents in the United States are employed in jobs that expose them to challenging working conditions, including low wages, limited benefits, and unstable and unpredictable work schedules. A growing body of literature has documented the harmful impacts of unstable and unpredictable scheduling on workers and their families. We focus on the connection between parental exposure to such unstable and unpredictable scheduling practices and children’s asthma management. Although not all risk factors are fully understood, researchers have linked genetics, environment, and social conditions to adequate asthma control. To this end, we estimate the association between parental exposure to unstable and unpredictable scheduling practices in the service sector and children’s asthma control.

**Methods:**

This study draws on survey data from the Shift Project about children under the age of 10 whose parents are employed in the retail and service sectors (*N*=2,994). Using a series of OLS regressions with adjustment for confounders, we examine the association between parental exposure to unstable and unpredictable work schedules and their children’s asthma management. We also perform analyses to study whether parental health and well-being mediates this relationship.

**Results:**

We document that parental exposure to unstable and unpredictable scheduling practices heightens children’s risk of wheezing episodes and emergency department visits for asthma, with effect sizes of between 0.3 and 0.5 of a SD. The association between parental exposure to work schedule unpredictability and wheezing is significantly mediated by parental work-life conflict and well-being.

**Discussion:**

This study identifies a previously unexplored factor, parental exposure to unstable and unpredictable work schedules, that shapes children’s asthma management and adds to growing evidence on the consequences of schedule instability for the health and well-being of both workers *and* their children.

**Conclusions:**

Ultimately, unstable work schedules among parents may further contribute to the already unequal distribution of asthma in children, ultimately exacerbating broader health inequalities in the United States.

**Supplementary Information:**

The online version contains supplementary material available at 10.1186/s12889-026-26274-y.

## Background

Asthma is a chronic lung condition that affects approximately 20 million adults and five million children in the United States [1]. Yet, the burden of the disease is unequally distributed across the population: children affected by poverty are more likely to develop and be negatively affected by asthma due to poor quality housing and the cost of medication, among other influences [2, 3]. Asthma, especially when poorly controlled, has lasting negative consequences through childhood and beyond, including sitting out from physical education class to missing school entirely. While risk factors for poor asthma control include environmental factors like air quality, [4] social conditions that influence stress and the capacity to respond to flare-ups can play an equally important role [5], Accordingly, research has suggested that parental involvement is critical to controlling asthma symptoms because parents must recognize wheezing in, give inhalers to, and make appointments for their children [6].

In this paper, we focus on the ability of parents in the retail and service sectors, who often have precarious work schedules, to effectively manage their children’s asthma. For the 20% of the labor force employed in these areas [7], a regular day shift is far from the norm. Instead, workers often face “just-in-time” schedules that are unstable and unpredictable, varying from day-to-day and week-to-week, often with little advance notice [8, 9]. As a result, workers who are exposed to such schedules report higher levels of psychological distress and unhappiness as well as significantly greater work-life conflict when compared to workers at the same firms with more stable and predictable schedules [10, 11].

**Fig. 1:**
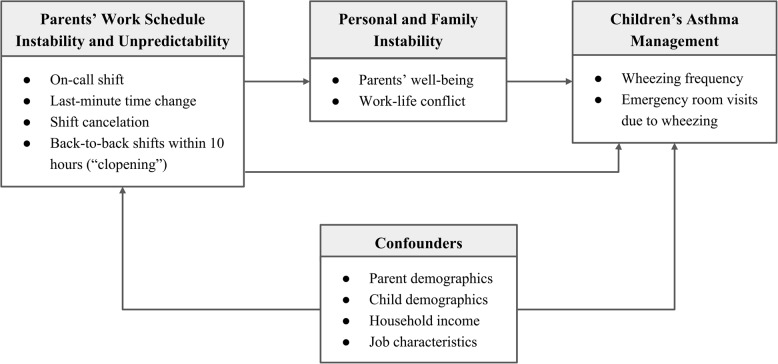
Expected Associations between Parental Exposure to Schedule Instability and Unpredictability, Mediating Factors, and Asthma Control

At the same time, psychologists and health researchers have documented the negative relationship between parental stress levels and asthma control, particularly for young children [12–14]. Against this backdrop, *we study how parents’ precarious work schedules influence their children’s asthma control, and whether this relationship is mediated by the psychological burden, stress, and work-life conflict created by parental exposure to unpredictable schedules.* Figure 1 provides a path diagram that illustrates the hypothesized relationships between scheduling (the left-hand box), which is our key independent variable and children’s asthma control (the right-hand box), while attending to potential confounders (lower box). We examine how parental and family moderators, including parents’ well-being and work-life conflict (center box) act as mediators in this relationship.

These inequities are of concern because children with uncontrolled asthma may experience a greater risk to their physical well-being in the short and long terms, potentially exacerbating intergenerational inequality. Our analysis is the first to examine how parental exposure to precarious scheduling affects children’s asthma control and joins a sparse body of prior research that has focused on parental exposure to unstable and unpredictable scheduling and children’s health and well-being more generally [15–17].

## Methods

### Data

We draw on data collected by the Shift Project, which has administered national web-based surveys to workers across the retail and service sectors since 2016. These data have been collected in twice annual repeated cross-sections that are fielded in the spring (March - June) and fall (August - October). Using Facebook’s advertising platform, the Shift Project targets workers at 130 of the largest firms in the retail and food service sectors, including retail apparel, grocery, hardware, big box, pharmacy, fast food, and casual dining subsectors. Study advertisements appeared in the Facebook desktop or mobile news- feeds or Instagram accounts of targeted users.

Users who clicked on the link in the advertisement were redirected to an online survey hosted through the Qualtrics platform. Respondents who completed the survey and provided contact information were entered into a drawing for an iPad. Approximately 1.2% of advertisement displays yielded survey data. Although these response rates are lower than obtained in many probability-sample phone surveys, a large sample of working parents employed in the service sector would be difficult if not impossible to reach through traditional methods given the absence of an appropriate sampling frame. Schneider and Harknett [18] describe elsewhere in greater detail the data collection procedures for the Shift Project. A key additional benefit of the Shift Project data is that it is the only source of large-scale survey data that includes detailed measures of parental work scheduling and children’s health outcomes.

The Shift Project method of collecting data results in a strategically targeted, non- probability sample, raising concerns about representativeness and bias. Although the use of Facebook as a sampling frame will largely exclude workers without internet access and who are not active on Facebook or Instagram, [19] recent estimates suggest that 84% of working adults aged 18–50 years are active on Facebook or Instagram, and, critically, this share does not vary by household income [20]. Zhang and colleagues [21] find a high degree of comparability between respondents drawn using a similar approach through Facebook and the American Community Survey in terms of veteran status, homeownership, and nativity, giving more confidence to our sampling method. Prior validation work from the Shift Project shows that univariate distributions of work scheduling exposures and bivariate associations are similar in the Shift data as in the Current Population Survey and National Longitudinal Survey of Youth-1997.

To ensure further generalizability, we post-stratify and weight the Shift Project parent sub-sample along the dimensions of race/ethnicity, age, gender, and education to the “gold-standard” of parents in the same set of occupations and industries who are captured in the 2008-2017 American Community Survey. These techniques are commonly used in forecasting and public opinion research to align non-probability samples with population characteristics [22, 23].To ensure that the representation of parents by employer matches the relative employee sizes of each employer in the data, we calculate total employment at each of the firms in the data from the Reference USA database, collapsing establishment-level employment from 365,294 establishments into firm-level counts [24]. Consistent with prior work from the Shift Project [8, 18, 25, 26], we apply these firm weights to all analyses. After multiple imputation for item non-response, our analysis sample is composed of 2,994 parents with a child under the age of 10 who were surveyed between the fall of 2018 and spring of 2020 and asked a set of detailed questions about children’s asthma control. We also draw on a subset of these 448 respondents who reported that their children either had asthma or episodes of wheezing, and were asked about emergency department (ED) visits for those medical problems. The study was approved by the authors’ institutional review board.

### Measures

#### Asthma control

Asthma control or management refers to the measures patients or caregivers take to treat asthma symptoms, including wheezing, tightness in the chest, and shortness of breath [27]. As such, we construct two dependent variables from the Shift Project survey data to represent the severity of asthma: wheezing incidents and occurrence of ED visits. First, respondents are asked to report how often over the past 12 months their child had a wheezing attack that made it difficult for the child to catch their breath. Possible responses include “Never,” “Fewer than three times *all together,*” “4–10 times *all together,*” “1–2 times *per week,*” “More than once *per week,*” and “Every day.” We top code this response at the 99^th^ percentile, which is the category “1–2 times per week.” We model this outcome as a continuous variable as well as a dichotomous variable that differentiates between those with no wheezing episodes and those with at least one episode. Second, respondents are asked to report how many days (ranging from zero to 365 days) in the previous 12 months their children needed to go to a hospital due to asthma or wheezing. Again, outlier values were top-coded to the 99th percentile. We model this outcome as both a continuous variable and as a dichotomous variable that differentiates those with no ED visits from those with at least one visit. These measures both rely on parent reports of wheezing. While we refer to the outcomes as “wheezing” and “ED visits” in the following discussion for brevity of phrasing, it is important to keep in mind that these are parental reports rather than direct measurements.

#### Work schedule instability and unpredictability

We measure our key independent variables, parents’ schedule unpredictability and instability, with four indicators. These binary measures are each coded to “1” and “0” according to whether 1) they worked any “on-call” shifts in the prior month, 2) reported at least one canceled shift in the prior month, 3) their shift timing was changed by their employer at least once in the past month, and 4) whether they worked any “clopening” shifts, defined as a shift that closes the establishment followed by a shift that opens the establishment with fewer than 10 hours in between. We also create an additive scale that ranges from “0” (no exposure to on- call shifts, canceled shifts, last-minute timing changes, or clopening shifts) to “4” (experienced all four practices at least once in the prior month) to measure the degree of scheduling instability [25].

#### Parental well-being and work-life conflict

To test our hypothesis, we examine if these associations between scheduling stability and asthma control indicators are mediated by parental well-being and work-life conflict. We describe the components of each of the scales that we construct for these two mediators, calculated using the alpha command in Stata and we show the Cronbach’s alpha for each scale [28]. We measure parental well-being to capture the pathway by which unpredictable work schedules negatively affect asthma control by increasing parental stress and depressing parental mood. Specifically, we construct a reliability scale composed of three sets of binary well-being indicators (α = 0.61): whether parents reported being “pretty” or “very” happy as opposed to “not too” happy; reported “good” or “very good” sleep quality over the past month; and scored below 12 on the K-6 scale of psychological distress [29] with reference to affect over the past month, the generally accepted cut-off for significant psychological distress. We also create a reliability scale for work-life conflict from three additional sets of binary indicators, including reported the ability to handle personal matters while at work, that their shift causes extra stress for themselves and family, and that they have enough flexibility in their schedule to handle family needs (α = 0.78) [30,31].

#### Control variables

We control for a set of demographic and job quality characteristics that might confound any association between parental exposure to unstable and unpredictable work schedules and control of children’s asthma. We control for parental age, race/ethnicity (white, non-Hispanic; Black, non-Hispanic; Asian, non-Hispanic; Hispanic; or other race/ethnicity), gender, educational attainment, parental school enrollment, marital status (married and living with spouse, unmarried and living with partner, not living with spouse or partner), whether the parent is a manager, union member, household income, usual weekly work hours, and involuntary part time work (defined as working fewer than 35 hours, but wanting more hours). We additionally control for the focal child’s age and gender as well as for the total number of children in the household. Lastly, we include a set of fixed effects for year and month to account for seasonal variation in asthma.

### Analytical methods

To estimate the association between parental exposure to scheduling unpredictability and children’s asthma control, we regress each dependent variable—wheezing frequency, any wheezing, number of ED visits, and any ED visits for asthma or wheezing — separately on each of the four scheduling variables – being on-call, having a canceled shift, having a last-minute timing change, or working a clopening, with the controls noted above. This yields 16 models. We estimate an ordinary least squares (OLS) regression model because we theorize that each additional exposure to scheduling instability (i.e., increase in the instability scale) has an additive effect on indicators of asthma management. We also employ an OLS model for the dichotomous outcomes, which facilitates the mediation analyses. Finally, we use the -khb- routine [32] in Stata to estimate if parental work-life conflict and psychological well-being significantly mediate any association between parental exposure to work schedule unpredictability and instability and child asthma control.

## Results

### Parental schedule unpredictability/instability and children’s asthma control

Table 1 contains the results for the regressions of both wheezing and ED visits on each of the four individual measures of schedule instability. We find modest support for our hypothesis that exposure to scheduling instability is associated with an increased risk of children experiencing wheezing. For wheezing frequency (Model 1), the association with each exposure is generally in the anticipated direction but is only marginally significant for last-minute timing changes (8 percentage points, *p*=.089). We find more consistent associations between schedule instability and the likelihood that the child will experience any wheezing (Model 2). Two out of the four theorized scheduling exposures are associated with significant increases in the likelihood that children will experience any wheezing: 6 points (*p* =.012) for last-minute timing changes, and 5 points (*p* =.032) for clopening shifts, with canceled shifts associated at the *p* < 0.10 level (B = 0.08; *p*=.089).

**Table 1:**
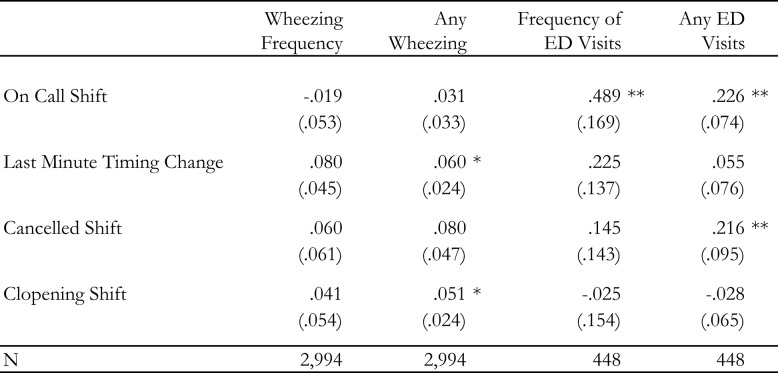
Parental exposure to schedule instability and unpredictability and reported asthma control

OLS regression coefficients (*p*-values) are presented for each estimate. Model covariates included parental age, race, gender, marital status, educational attainment, household income, weekly hours, as well as whether they are a manager, a member of a union, enrolled in school themselves, and engaging involuntary part-time work. We also include controls for the focal child’s age, gender, and number of children in the household. Each model contains a set of fixed effects for year and month.

Statistical significance levels are indicated as follows:

*p value < 0.05

**p value < 0.01

***p value < 0.001

We also find evidence that parental exposure to unstable and unpredictable scheduling may raise both the frequency (Model 3) and likelihood (Model 4) of ED visits for asthma or wheezing. For the Frequency of ED visits (Model 3), working at least one on-call shift in the prior month was associated with.49 (*p*=.004) more ED visits. However, the coefficients on the remaining types of scheduling exposures—last-minute timing changes, canceled and clopening shifts—are not statistically significant predictors of the frequency of ED visits. For any ED visits (Model 4), working on-call (23 points, *p*=.002) and canceled shifts (22 points, *p* =.024) are associated with a significant increase in the likelihood of the child ED visit for asthma or wheezing episodes.

In Table 2, we show the association between the degree of parental exposure to unstable and unpredictable work schedules and each outcome, using both a categorical specification of the instability scale and a continuous specification. For wheezing frequency (Model 1), we see relatively little evidence of positive association between greater parental exposure to unpredictable scheduling and frequency of wheezing, whether using a categorical measure of scheduling exposures or a continuous measure. In contrast, Model 2 shows that there is a strong and significant association between schedule instability and our dichotomous measure of any wheezing. Children whose parents were exposed to more than two types of instability are more likely to have wheezing than those whose parents have stable schedules by 9.2 points (*p*=.009 for) two exposures, and by 9.2 points (*p*=.016) for three or more exposures. We also find a statistically significant association between the continuous measure of schedule instability and any wheezing (Beta = 0.037, *p* <.01). Taking predicted probabilities and comparing children whose parents had no exposure to schedule instability against those whose parents had the most exposure, we estimate a difference of a third of a standard deviation in the outcome.

**Table 2:**
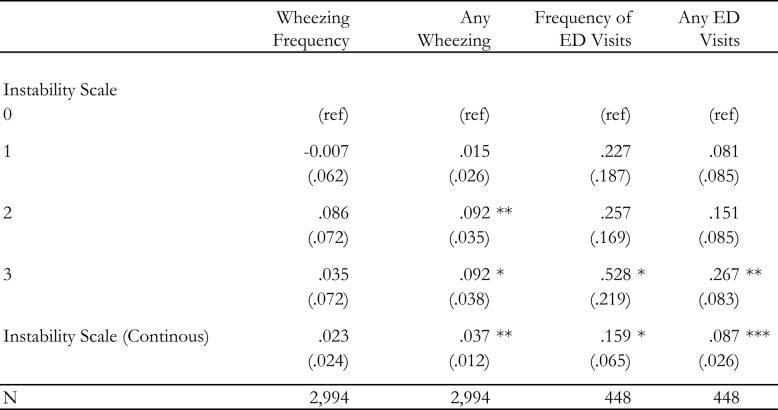
Work instability scale and reported asthma control

OLS regression coefficients (*p*-values) are presented for each estimate. Model covariates included parental age, race, gender, marital status, educational attainment, household income, weekly hours, as well as whether they are a manager, a member of a union, enrolled in school themselves, and engaging involuntary part-time work. We also include controls for the focal child’s age, gender, and number of children in the household. Each model contains a set of fixed effects for year and month. The sources of scheduling instability refer to on-call work, last-minute time changes, cancellations, and clopening shifts.

Statistical significance levels are indicated as follows:

*p value < 0.05

**p value < 0.01

***p value < 0.001

*ref* reference category

For ED visits, in Model 3, we also see a significant association between exposure to three or more sources of instability and the frequency of visits (B =0.53; *p*=.016) as well as a significant association between the continuous measure of schedule instability and the frequency of visits (B = 0.159; *p* <.05). Here, again taking predicted values from the model and contrasting the lowest (instability scale = 0) and highest (instability scale = 3) exposures, we find a difference of.46 SDs in the outcome. We similarly find that children whose parents had more types of exposure were significantly more likely to have had any ED visit (Model 4): a 27-point (*p*=<.001) increase for those with three or more types of exposure versus those with stable and predictable schedules, and 8.7 points for our continuous measure (*p*=<.001). Taking predicted values of the outcome at the least and greatest levels of exposure to schedule instability, we find a difference of.54 SD of the outcome.

### Mediation analyses

Our mediation analysis, reported in Table 3, supports the hypothesis that the stress associated with parents’ unpredictable schedules limits their ability to effectively control their children’s asthma. This table decomposes the portion of the “total association” between scheduling and our outcomes into the “Direct (unmediated) Association” and the “Indirect (mediated) Association.” The latter reflects the role of our two mediators, parental well-being and work-life conflict in mediating the association between parental scheduling exposures and our outcomes.

We find that, especially for any wheezing (Model 2), work-life conflict and parental well-being mediate the association between two and three or more sources of schedule instability. For two sources, these scales mediate approximately 15% of the association (0.014 (the mediated component)/0.092 (the total association)) (*p*=.087); for three or more sources, these measures mediate about a 30% of the association (0.027 (the mediated component)/0.092 (the total association)) (*p*=.047). In contrast, we find no evidence of any significant mediation of the association between schedule instability and frequency of ED (Model 3) visits or any ED visits (Model 4).

**Table 3:**
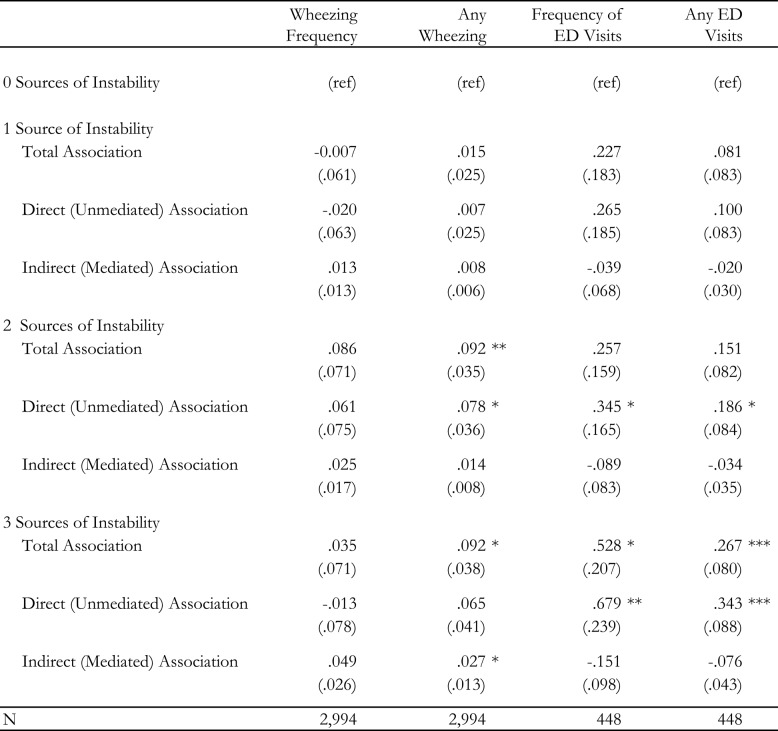
Mediation of association between schedule instability and reported asthma control by work-life conflict and parental well-being

Coefficients from the -khb- decomposition (*p*-values) are presented for each estimate. Model covariates included parental age, race, gender, marital status, educational attainment, household income, weekly hours, as well as whether they are a manager, a member of a union, enrolled in school themselves, and engaging involuntary part-time work. We also include controls for the focal child’s age, gender, and number of children in the household. Each model contains a set of fixed effects for year and month.

Statistical significance levels are indicated as follows:

*p value < 0.05

**p value < 0.01

***p value < 0.001

*ref* reference category

## Conclusions

Work schedule instability and unpredictability have become widespread in the service sector [8, 9]. Prior work has focused on the negative consequences of such precarious scheduling for worker economic outcomes as well as for workers’ health and well-being [8, 10]. However, these scheduling practices also have the potential to negatively affect children’s health and well-being through multiple pathways. The negative effects of work schedule instability on parental happiness, psychological distress, and work-life conflict are likely to spillover and negatively affect children’s outcomes. Yet, very little research has examined how parental exposure to precarious scheduling may affect the well-being of their children, with the only prior studies we are aware of focused on children’s sleep quality and children’s internalizing and externalizing behavior [15–17].

We hypothesized that parental exposure to precarious scheduling practices would negatively affect children’s asthma control. Children, particularly younger children, need help controlling their asthma, leaving parents to make appointments, give inhalers, and/or notice wheezing [33–35]. Asthma can be difficult to manage, as parents must be consistently attentive to their children’s symptoms and triggers, as well as ensure that schools and other caregivers are aware of their children’s specific needs [36, 37]. Stress and other psychological burdens, however, may get in the way of asthma control [38]. For example, primary caregivers with depressive symptoms may compromise the steps in their children’s asthma control plan by failing to avoid triggers and properly using medications which may make wheezing more likely [39, 40].

Our results provide the first evidence of the association between parental exposure to work schedule instability and unpredictability and children’s asthma control. We find that children whose parents are exposed to more sources of work schedule instability and unpredictability (on-call shifts, last minute timing changes, canceled shifts, and clopening) are more likely to experience wheezing and more likely to have ED visits. In the case of any wheezing, these associations are significantly mediated by parental well-being and work-life conflict.

This work is also subject to several important limitations. First, the Shift Project survey provides the only existing data that combines a large sample of hourly service sector workers with detailed measurement of work schedule stability and unpredictability alongside children’s asthma control. However, it is a non-probability sample, with a low-response rate (1.2%) which may reduce generalizability. Second, our analyses are observational and while we include a rich set of controls, we cannot rule out the possibility of omitted variable bias. Our hypotheses are theory-driven and focused on a small set of pre-specified relationships, and we therefore do not apply formal multiple-testing corrections. In line with recent methodological discussions in the public health and medical literature, such adjustments would be overly conservative in this context and heighten the risk obscuring policy-relevant associations between scheduling instability and child health [41–44]. Finally, our measures of children’s asthma control are reported by parents and are not based on direct observation. Parents exposed to unstable and unpredictable schedules themselves have lower well-being [8–10] and this could be associated with over (or under) reporting of asthma control. Further, our measurement of marital status, which may proxy for parental availability, does not capture heterogeneity in the involvement of non-coresidential parents [45]. Accordingly, we emphasize that these findings should be interpreted as descriptive and exploratory rather than as causal.

In this paper, we draw on new data from the Shift Project to study the association between parental work schedules and children’s asthma control. We find support for our hypothesis that exposure to scheduling instability hinders parents’ ability to adequately control their children’s asthma. This research is of particular importance because it addresses the extent to which unstable work schedules is correlated with poorer health for both adults and children [46, 47]. By leveraging the unique combination of detailed measures of parental work scheduling and of asthma control, we are able to shed new light on this important dynamic. Our results point to the importance of parental exposure to unstable and unpredictable schedules as a source of childhood disadvantage. The COVID-19 pandemic brings new urgency to this issue as service sector workers are lauded as heroes, but appear to continue to contend with poor job quality and disrupted family routines. Future work might explore the relationship between scheduling instability and other measures of asthma control that we were not able to capture, such as medication use and doctor check-ups. This work is focused on workers who are exposed to both unstable and unpredictable schedules and to low wages. This limits the external validity of the research and future work could fruitfully examine if exposure to challenging work schedules in higher compensation occupations, such as in health care, have similar negative consequences or if other social advantages might mitigate such harms.

## Supplementary Information

Supplementary Material 1.

## Data Availability

The data and code are available from the authors upon request for the purposes of replication.

## Abbreviations

OLS: Ordinary least squares
ED: Emergency Department
K-6: Kessler 6 Scale
