# Supplementary material for: Parents’ precarious work schedules and children’s asthma management
Source: BMC Public Health. 2026 Feb 9;26:880. doi: 10.1186/s12889-026-26274-y (PMC12983562; doi:10.1186/s12889-026-26274-y)
Supplement: Supplementary file 1 — Supplementary Material 1. [file 12889_2026_26274_MOESM1_ESM.pdf]

STROBE Statement—checklist of items that should be included in reports of observational studies

	Item No.	Recommendation	Page No.	Relevant text from manuscript
**Title and abstract**	1	(*a*) Indicate the study's design with a commonly used term in the title or the abstract	2	We estimate the association between parental exposure to unstable and unpredictable scheduling practices in the service sector and children's asthma control.
		(*b*) Provide in the abstract an informative and balanced summary of what was done and what was found	2	**Methods** This study draws on survey data from the Shift Project about children under the age of 10 whose parents are employed in the retail and service sectors (N=2,994). Using a series of OLS regressions with confounders, we examine the association between parental exposure to unstable and unpredictable work schedules and their children's asthma management. We also perform analyses to study whether parental health and well-being mediates this relationship. **Results** We document that parental exposure to unstable and unpredictable scheduling practices heightens children's risk of wheezing episodes and emergency department visits for asthma, with effect sizes of between 0.3 and 0.5 of a SD. The association between parental exposure to work schedule unpredictability and wheezing is significantly mediated by parental work-life conflict and well-being.
**Introduction**				
Background/rationale	2	Explain the scientific background and rationale for the investigation being reported	4,5	These inequities are of concern because children with uncontrolled asthma may experience a greater risk to their physical well-being in the short and long terms, potentially exacerbating intergenerational inequality. Our analysis is the first to examine how parental exposure to precarious scheduling affects children's asthma control and joins a sparse body of prior research that has focused on parental exposure to unstable and unpredictable scheduling and children's health and well-being more generally.
Objectives	3	State specific objectives, including any prespecified hypotheses	4	Against this backdrop, we study how parents' precarious work schedules influence their children's asthma control, and whether this relationship is mediated by the psychological burden, stress, and work-life conflict created by parental exposure to unpredictable schedules. Figure 1 visualizes our hypotheses (p. 4)
**Methods**				

Study design	4	Present key elements of study design early in the paper	5-9	We draw on data collected by the Shift Project, which has administered national web-based surveys to workers across the retail and service sectors since 2016 (p. 5). To estimate the association between parental exposure to scheduling unpredictability and children's asthma control, we regress each dependent variable—wheezing frequency, any wheezing, number of ED visits, and any ED visits for asthma or wheezing — separately on each of the four scheduling variables – being on-call, having a canceled shift, having a last-minute timing change, or working a clopening, with the controls noted above (p. 9).
Setting	5	Describe the setting, locations, and relevant dates, including periods of recruitment, exposure, follow-up, and data collection	5	We draw on data collected by the Shift Project, which has administered national web-based surveys to workers across the retail and service sectors since 2016. These data have been collected in twice annual repeated cross-sections that are fielded in the spring (March - June) and fall (August - October). Using Facebook's advertising platform, the Shift Project targets workers at 130 of the largest firms in the retail and food service sectors, including retail apparel, grocery, hardware, big box, pharmacy, fast food, and casual dining subsectors. Study advertisements appeared in the Facebook desktop or mobile news- feeds or Instagram accounts of targeted users.
Participants	6	(*a*) *Cohort study*—Give the eligibility criteria, and the sources and methods of selection of participants. Describe methods of follow-up *Case-control study*—Give the eligibility criteria, and the sources and methods of case ascertainment and control selection.	5	Using Facebook's advertising platform, the Shift Project targets workers at 130 of the largest firms in the retail and food service sectors, including retail apparel, grocery, hardware, big box, pharmacy, fast food, and casual dining subsectors. Study advertisements appeared in the Facebook desktop or mobile news-feeds or Instagram accounts of targeted users. Users who clicked on the link in the advertisement were redirected to an online survey hosted through the Qualtrics platform.

		Give the rationale for the choice of cases and controls *Cross-sectional study*—Give the eligibility criteria, and the sources and methods of selection of participants		
		(*b*) *Cohort study*—For matched studies, give matching criteria and number of exposed and unexposed *Case-control study*—For matched studies, give matching criteria and the number of controls per case	n/a	n/a
Variables	7	Clearly define all outcomes, exposures, predictors, potential confounders, and effect modifiers.	7-9	Asthma control or management refers to the measures patients or caregivers take to treat asthma symptoms, including wheezing, tightness in the chest, and shortness of breath. 25 As such, we construct two dependent variables from the Shift Project survey data to represent the severity of asthma: wheezing incidents and occurrence of ED visits. First, respondents are asked to report how often in over the past 12 months their child had a wheezing attack that made it difficult for the child to catch their breath. Possible responses include "Never," "Fewer than three times all together," "4-10 times all together," "1-2 times per week," "More than once per week," and "Every day." We top code this response at the 99th

		Give diagnostic criteria, if applicable		percentile, which is the category "1-2 times per week." We model this outcome as a continuous variable as well as a dichotomous variable that differentiates between those with no wheezing episodes and those with at least one episode. Second, respondents are asked to report how many days (ranging from zero to 365 days) in the previous 12 months their children needed to go to a hospital due to asthma or wheezing(7). We measure our key independent variables, parents' schedule unpredictability and instability, with four indicators. These binary measures are each coded to "1" and "0" according to whether 1) they worked any "on-call" shifts in the prior month, 2) reported at least one canceled shift in the prior month, 3) their shift timing was changed by their employer at least once in the past month, and 4) whether they worked any "clopening" shifts, defined as a shift that closes the establishment followed by a shift that opens the establishment with fewer than 10 hours in between (p. 8). "We measure parental well-being to capture the pathway by which unpredictable work schedules negatively affect asthma control by increasing parental stress and depressing parental mood. Specifically, we construct a reliability scale composed of three sets of binary well-being indicators ($\alpha = 0.61$): whether parents reported being "pretty" or "very" happy as opposed to "not too" happy; reported "good" or "very good" sleep quality over the past month; and scored below 12 on the K-6 scale of psychological distress 28 with reference to affect over the past month, the generally accepted cut-off for significant psychological distress" (p. 8). We control for a set of demographic and job quality characteristics that might confound any association between parental exposure to unstable and unpredictable work schedules and control of children's asthma. We control for parental age, race/ethnicity (white, non-Hispanic; Black, non-Hispanic; Asian, non-Hispanic; Hispanic; or other race/ethnicity), gender, educational attainment, parental school enrollment, marital status (married and living with spouse, unmarried and living with partner, not living with spouse or partner), whether the parent is a manager, union member, household income, usual weekly work hours, and involuntary part time work (defined as working fewer than 35 hours, but wanting more hours). We additionally control for the focal child's age and gender as well as for the total number of children in the household. Lastly, we include a set of fixed effects for year and month to account for seasonal variation in asthma (p. 9).
Data sources/ measurement	8*	For each variable of interest, give sources of data and details of methods of assessment	7-9	See above.

		(measurement). Describe comparability of assessment methods if there is more than one group		
Bias	9	Describe any efforts to address potential sources of bias	6	The Shift Project method of collecting data results in a strategically targeted, non- probability sample, raising concerns about representativeness and bias. Although the use of Facebook as a sampling frame will largely exclude workers without internet access and who are not active on Facebook or Instagram, 19 recent estimates suggest that 84% of working adults aged 18-50 years are active on Facebook or Instagram, and, critically, this share does not vary by household income…To ensure further generalizability, we post-stratify and weight 22 the Shift Project parent sub-sample along the dimensions of race/ethnicity, age, gender, and education to the "gold- standard" of parents in the same set of occupations and industries who are captured in the 2008- 2017 American Community Survey. To ensure that the representation of parents by employer matches the relative employee sizes of each employer in the data, we calculate total employment at each of the firms in the data from the Reference USA database, collapsing establishment-level employment from 365,294 establishments into firm-level counts.
Study size	10	Explain how the study size was arrived at	6	After multiple imputation for item non-response, our analysis sample is composed of 2,994 parents with a child under the age of 10 who were surveyed between the fall of 2018 and spring of 2020 and asked a set of detailed questions about children's asthma control.
Quantitative variables	11	Explain how quantitative variables were handled in the analyses. If applicable, describe which groupings were chosen and why	9	To estimate the association between parental exposure to scheduling unpredictability and children's asthma control, we regress each dependent variable—wheezing frequency, any wheezing, number of ED visits, and any ED visits for asthma or wheezing — separately on each of the four scheduling variables – being on-call, having a canceled shift, having a last-minute timing change, or working a clopening, with the controls noted above. This yields 16 models. We estimate an ordinary least squares (OLS) regression model because we theorize that each additional exposure to scheduling instability (i.e., increase in the instability scale) has an additive effect on indicators of asthma management.
Statistical methods	12	(*a*) Describe all statistical methods,	9-10	See above.

		including those used to control for confounding		
		(*b*) Describe any methods used to examine subgroups and interactions	n/a	n/a
		(*c*) Explain how missing data were addressed	5	Respondents who completed the survey and provided contact information were entered into a drawing for an iPad. Approximately 1.2% of advertisement displays yielded survey data. Although these response rates are lower than obtained in many probability-sample phone surveys, a large sample of working parents employed in the service sector would be difficult if not impossible to reach through traditional methods given the absence of an appropriate sampling frame.
		(*d*) *Cohort study*—If applicable, explain how loss to follow-up was addressed *Case-control study*—If applicable, explain how matching of cases and controls was addressed *Cross-sectional study*—If applicable, describe analytical methods taking account of sampling strategy	6	To ensure further generalizability, we post-stratify and weight the Shift Project parent sub-sample along the dimensions of race/ethnicity, age, gender, and education to the "gold- standard" of parents in the same set of occupations and industries who are captured in the 2008- 2017 American Community Survey. To ensure that the representation of parents by employer matches the relative employee sizes of each employer in the data, we calculate total employment at each of the firms in the data from the Reference USA database, collapsing establishment-level employment from 365,294 establishments into firm-level counts. We apply these firm weights to all analyses.
		€ Describe any sensitivity analyses	n/a	n/a
**Results**				
Participants	13*	(a) Report numbers of individuals at each stage of study—eg numbers potentially eligible,	6,7	After multiple imputation for item non-response, our analysis sample is composed of 2,994 parents with a child under the age of 10 who were surveyed between the fall of 2018 and spring of 2020 and asked a set of detailed questions about children's asthma control. We also draw on a subset of these 448 respondents who reported that their children either had 7 asthma or episodes of wheezing, and were

		examined for eligibility, confirmed eligible, included in the study, completing follow-up, and analysed		asked about emergency department (ED) visits for those medical problems. We note that Schneider and Harknett describe elsewhere in greater detail the data collection procedures for the Shift Project (p. 5).
		(b) Give reasons for non-participation at each stage	5-7	See above.
		(c) Consider use of a flow diagram	5-7	See above.
Descriptive data	14*	(a) Give characteristics of study participants (eg demographic, clinical, social) and information on exposures and potential confounders	5, 9	Using Facebook's advertising platform, the Shift Project targets workers at 130 of the largest firms in the retail and food service sectors, including retail apparel, grocery, hardware, big box, pharmacy, fast food, and casual dining subsectors (p. 5). We control for a set of demographic and job quality characteristics that might confound any association between parental exposure to unstable and unpredictable work schedules and control of children's asthma. We control for parental age, race/ethnicity (white, non-Hispanic; Black, non-Hispanic; Asian, non-Hispanic; Hispanic; or other race/ethnicity), gender, educational attainment, parental school enrollment, marital status (married and living with spouse, unmarried and living with partner, not living with spouse or partner), whether the parent is a manager, union member, household income, usual weekly work hours, and involuntary part time work (defined as working fewer than 35 hours, but wanting more hours).<br>We additionally control for the focal child's age and gender as well as for the total number of children in the household (p. 9).
		(b) Indicate number of participants with missing data for each variable of interest	6-7	See 13.
		© *Cohort study*—Summarise follow-up time (eg, average and total amount)	n/a	n/a

Outcome data	15*	*Cohort study*—Report numbers of outcome events or summary measures over time	n/a	n/a
		Case-control study—Report numbers in each exposure category, or summary measures of exposure	n/a	n/a
		Cross-sectional study—Report numbers of outcome events or summary measures	11-13	Tables 1 and 2 provide the total number of respondents for each measure.
Main results	16	(*a*) Give unadjusted estimates and, if applicable, confounder-adjusted estimates and their precision (eg, 95% confidence interval). Make clear which confounders were adjusted for and why they were included	10-11	Table 1 contains the results for the regressions of both wheezing and ED visits per month on each of the four individual measures of schedule instability. We find modest support for our hypothesis that exposure to scheduling instability is associated with an increased risk of children experiencing wheezing. For wheezing frequency (Model 1), the association with each exposure is generally in the anticipated direction but is only marginally significant for last-minute timing changes (8 percentage points, p=.089). We find more consistent associations between schedule instability and the likelihood that the child will experience any wheezing (Model 2). Two out of the four theorized scheduling exposures are associated with significant increases in the likelihood that children will experience any wheezing: 6 points (p =.012) for last-minute timing changes, and 5 points (p =.032) for clopening shifts, with canceled shifts associated at the p < 0.10 level (B = 0.08; p=.089).<br>We also find evidence that parental exposure to unstable and unpredictable scheduling may raise both the frequency (Model 3) and likelihood (Model 4) of ED visits for asthma or wheezing. For the Frequency of ED visits (Model 3), working at least one on-call shift in the prior month was associated with .49 (p=.004) more ED visits. However, the coefficients on the remaining types of scheduling exposure—last-minute timing changes, canceled and clopening shifts—are not statistically significant predictors of the frequency of ED visits. For any ED visits (Model 4), working on-call (23 points, p=.002) and canceled shifts (22 points, p =.024) are

				associated with a significant increase in the likelihood of the child requiring an ED visit for asthma or wheezing episodes.
		(*b*) Report category boundaries when continuous variables were categorized	7-8	As such, we construct two dependent variables from the Shift Project survey data to represent the severity of asthma: wheezing incidents and occurrence of ED visits. First, respondents are asked to report how often over the past 12 months their child had a wheezing attack that made it difficult for the child to catch their breath. Possible responses include "Never," "Fewer than three times *all together,*" "4-10 times *all together,*" "1-2 times *per week,*" "More than once *per week,*" and "Every day." We top code this response at the 99th percentile, which is the category "1-2 times per week." We model this outcome as a continuous variable as well as a dichotomous variable that differentiates between those with no wheezing episodes and those with at least one episode. Second, respondents are asked to report how many days (ranging from zero to 365 days) in the previous 12 months their children needed to go to a hospital due to asthma or wheezing. Again, outlier values were top-coded to the 99th percentile. We model this outcome as both a continuous variable and as a dichotomous variable that differentiates those with no ED visits from those with at least one visit.<br>We also create an additive scale that ranges from "0" (no exposure to on- call shifts, canceled shifts, last minute timing changes, or clopening shifts) to "4" (experienced all four practices at least once in the prior month) to measure the degree of scheduling instability.
		(*c*) If relevant, consider translating estimates of relative risk into absolute risk for a meaningful time period	n/a	n/a
Other analyses	17	Report other analyses done—eg analyses of subgroups and interactions, and sensitivity analyses	13	Our mediation analysis, reported in Table 3, supports the hypothesis that the stress associated with parents' unpredictable schedules limits their ability to effectively control their children's asthma. This table decomposes the portion of the "total association" between scheduling and our outcomes into the "Direct (unmediated) Association" and the "Indirect (mediated) Association." The latter reflects the role of our two mediators, parental wellbeing and work-life conflict in mediating the association between parental scheduling exposures and our outcomes.<br>We find that, especially for any wheezing (Model 2), work-life conflict and parental well-being mediate the association between two and three or more sources of schedule instability. For two sources, these scales mediate approximately 15% of the association (0.014 (the mediated component) / 0.092 (the total association)) (p=.087); for three or more sources, these measures

				mediate about a 30% of the association (0.027 (the mediated component) / 0.092 (the total association)) (p=.047). In contrast, we find no evidence of any significant mediation of the association between schedule instability and frequency of ED (Model 3) visits or any ED visits (Model 4).
**Discussion**				
Key results	18	Summarise key results with reference to study objectives	15, 16	We hypothesized that parental exposure to precarious scheduling practices would negatively affect children's asthma control. Our results provide the first evidence of the association between parental exposure to work schedule instability and unpredictability and children's asthma control. We find that children whose parents are exposed to more sources of work schedule instability and unpredictability (on-call shifts, last minute timing changes, canceled shifts, and clopening) are more likely to experience wheezing and more likely to have ED visits. In the case of any wheezing, these associations are significantly mediated by parental well-being and work-life conflict.
Limitations	19	Discuss limitations of the study, taking into account sources of potential or imprecision. Discuss both direction and magnitude of any potential bias	16	However, it is a non-probability sample, with a low-response rate (1.2%) which may reduce generalizability. Additionally, our analyses are observational and while we include a rich set of controls, we cannot rule out the possibility of omitted variable bias. Finally, in terms of measurement, our measures of children's asthma control are reported by parents and are not based on direct observation.
Interpretation	20	Give a cautious overall interpretation of results considering objectives, limitations, multiplicity of analyses, results from similar studies, and other relevant evidence	16-17	In this paper, we draw on new data from the Shift Project to study the association between parental work schedules and children's asthma control. We find support for our hypothesis that exposure to scheduling instability hinders parents' ability to adequately control their children's asthma. This research is of particular importance because it addresses the extent to which unstable work schedules is correlated with poorer health for both adults and children. 40 , 41 By leveraging the unique combination of detailed measures of parental work scheduling and of asthma control, we are able to shed new light on this important dynamic. Our results point to the importance of parental exposure to unstable and unpredictable schedules as a source of childhood disadvantage. The COVID-19 pandemic brings new urgency to this issue as service sector workers are lauded as heroes, but appear to continue to contend with poor job quality and disrupted family routines. Future work might explore the relationship between scheduling instability and other measures of asthma control that we were not able to capture, such as medication use and doctor check-ups.

				We do, however, note that this work is subject to several important limitations. First, the Shift Project survey provides the only existing data that combines a large sample of hourly service sector workers with detailed measurement of work schedule stability and unpredictability alongside children's asthma control. However, it is a non-probability sample, with a low-response rate (1.2%) which may reduce generalizability. Second, our analyses are observational and while we include a rich set of controls, we cannot rule out the possibility of omitted variable bias. Our hypotheses are theory-driven and focused on a small set of pre-specified relationships, and we therefore do not apply formal multiple-testing corrections. In line with recent methodological discussions in the public health and medical literature, such adjustments would be overly conservative in this context and heighten the risk obscuring policy-relevant associations between scheduling instability and child health. Finally, our measures of children's asthma control are reported by parents and are not based on direct observation. Parents exposed to unstable and unpredictable schedules themselves have lower well-being and this could be associated with over (or under) reporting of asthma control. Further, our measurement of marital status, which may proxy for parental availability, does not capture heterogeneity in the involvement of non-coresidential parents. Accordingly, we emphasize that these findings should be interpreted as descriptive and exploratory rather than as causal (p. 17).
Generalisability	21	Discuss the generalisability (external validity) of the study results	17	This work is focused on workers who are exposed to both unstable and unpredictable schedules and to low wages. This limits the external validity of the research and future work could fruitfully examine if exposure to challenging work schedules in higher compensation occupations, such as in health care, have similar negative consequences or if other social advantages might mitigate such harms.
**Other information**				
Funding	22	Give the source of funding and the role of the funders for the present study and, if applicable, for the original study on which the present article is based	17	The authors received support from the W.T. Grant Foundation (188043), the National Institutes of Child Health and Human Development (R21HD091578), the Robert Wood Johnson Foundation (Award No. 74528), and the Bill and Melinda Gates Foundation (INV- 002665). Perez and Quinn received additional support from the National Institutes of Aging (R25AG047848) through the Cal-ADAR program at UC Berkeley.

*Give information separately for cases and controls in case-control studies and, if applicable, for exposed and unexposed groups in cohort and cross-sectional studies.

Note: An Explanation and Elaboration article discusses each checklist item and gives methodological background and published examples of transparent reporting. The STROBE checklist is best used in conjunction with this article (freely available on the Web sites of PLoS Medicine at http://www.plosmedicine.org/, Annals of Internal Medicine at http://www.annals.org/, and Epidemiology at http://www.epidem.com/). Information on the STROBE Initiative is available at www.strobe-statement.org.
